# As predicted by theory: choice and competition in a publicly funded and regulated regional health system yield improved access and cost control

**DOI:** 10.1186/s12913-021-06392-6

**Published:** 2021-05-01

**Authors:** Jonas Wohlin, Clara Fischer, Karin Solberg Carlsson, Sara Korlén, Pamela Mazzocato, Carl Savage, Holger Stalberg, Mats Brommels

**Affiliations:** 1 Accumbo AB, SE-39230 Kalmar, Sweden; 2 Medical Management Centre, Department of Learning, Informatics, Management and Ethics, Karolinska Institutet, SE-17177 Stockholm, Sweden; 3 Region Stockholm, SE-11221 Stockholm, Sweden

**Keywords:** Value-based health care, Bundled payment, New public management, Competition, Patient choice

## Abstract

**Background:**

New Public Management (NPM) has been widely used to introduce competition into public healthcare. Results have been mixed, and there has been much controversy about the appropriateness of a private sector-mimicking governance model in a public service. One voice in the debate suggested that rather than discussing whether competition is “good” or “bad” the emphasis should be on exploring the conditions for a successful implementation.

**Methods:**

We report a longitudinal case study of the introduction of patient choice and allowing private providers to enter a publicly funded market. Patients in need of hip or knee replacement surgery are allowed to choose provider, and those are paid a fixed reimbursement for the full care episode (bundled payment). Providers are financially accountable for complications. Data on number of patients, waiting lists and times, costs to the public purchaser, and complications were collected from public registries. Providers were interviewed at three points in time during a nine-year follow-up period. Time-series of the quantitative data were exhibited and the views of actors involved were explored in a thematic analysis of the interviews.

**Results:**

The policy goals of improving access to care and care quality while controlling total costs were achieved in a sustained way. Six themes were identified among actors interviewed and those were consistent over time. The design of the patient choice model was accepted, although all providers were discontent with the level of reimbursement. Providers felt that quality, timeliness of service and staff satisfaction had improved. Public and private providers differed in terms of patient-mix and developed different strategies to adjust to the reimbursement system. Private providers were more active in marketing and improving operation room efficiency. All providers intensified cooperation with referring physicians. Close attention was paid to following the rules set by the purchaser.

**Discussion and conclusions:**

The sustained cost control was an effect of bundled payment. What this study shows is that both public and private providers adhere long-term to regulations by a public purchaser that also controls entrance to the market. The compensation was fixed and led to competition on quality, as predicted by theory.

## Background

New Public Management (NPM) is by no means, “new”, as it was introduced in the 1980’s. It spread rapidly in North America and Western Europe, and became the recommended strategy to reorganise the public sector in the transition states of Central and Eastern Europe [1, 2]. Despite controversies, it is still seen as a vitalising strategy by governments and continues to be practised in public services worldwide, and can be identified in the background of other more recent reform initiatives, such as “Value-Based Health Care”.

The major criticism, as summarised by authors like Mongkol [3], Dunn and Miller [2], and Osborne et al. [1], is that the introduction of private sector management techniques and “managerialism”, focusing on performance management and output control, does not recognise the political, ethical, constitutional, and social dimensions of government. Public services have to meet complex needs, requiring a number of providers to interact and collaborate, emphasising the importance of an interorganisational perspective of public sector governance. NPM has an intra-organisational focus and lacks a systems perspective. Introducing markets and competition, the other central ingredient of NPM, leads to a disintegration of the public service system, and, it is claimed, will not be a suitable mechanism for allocating public resources [1].

In NPM, service providers compete for contracts or clients, or both. Early on, citizens’ right to choose their provider of public services was seen as a way to empower citizens and strengthen “participatory democracy” [4, 5]. In healthcare, critics emphasise that better educated and more affluent parts of the population make greater use of the opportunities to choose, which might increase health inequalities [6].

The experience of competition and choice in public service systems is vast, yet despite many empirical studies “the evidence is mixed and contested” [7]. Some indicate that “demand responds to quality and waiting times” and that competition between hospitals promotes quality [8]. Fotaki [9], on the other hand, concludes that patient choice in the UK had very limited impact on efficiency and quality with indications of negative effects on equity.

Goddard [7], in her scrutiny of the international debate, demonstrated how diverse and complex publicly funded health care systems are in terms of funding mechanisms, ownership of provider organisations, rules regulating competition, and regulations aiming at consumer protection, and controlling opportunistic or perverse provider behaviour. Her conclusion is that the “debate should be less about endlessly discussing whether it [competition] is ‘good’ or ‘bad’ and more about defining the circumstances under which it may work well…”.

Sweden was an early adopter of NPM in public services, including healthcare [6, 10]. Several public regional health authorities (counties) introduced a “purchaser-provider split” and internal markets already in the early 1990’s. Patients were allowed to choose their provider among public healthcare providers in order to stimulate competition, enhance efficiency, increase customer responsiveness, and improve access to services.

What would be the ideal conditions for choice and competition to work? Siciliani et al. [11] studied patient choice and provider competition in five European countries with publicly funded and regulated healthcare systems. Hospitals turned out to be increasingly reimbursed using fixed prices per patient episode, set by the funder, in order to stimulate competition on quality. The OECD project on payment systems studied practices in 15 member countries and found that bundled payments for episodes of care were introduced in order to improve care quality and control costs [12] .

Fotaki [6] discusses the fact that politicians in both the UK and Sweden, despite the modest results of the introduction of competition and choice in the 1990’s, have tended to reintroduce patient choice policies and defines that phenomenon as an “inability to unlearn” (meaning that politicians seem unwilling to change policy even if it does not lead to desired results). This raises the question whether there, after all, are some merits to these programmes. In Sweden, the reintroduction has been part of a conservative agenda, on both national and regional levels. Therefore, Sweden is a potentially informative case to study “circumstances under which” patient choice and competition “may work well” – or lead to consequences undermining intended policy goals.

This study utilises our ten years of close follow-up of one patient choice arrangement in the Stockholm Region, Sweden, i.e. elective hip and knee joint replacement surgery, popularly called “OrthoChoice”. The aim was to scrutinise this case especially how providers viewed and adjusted to competition and fixed reimbursements, and to explore whether the intended policy goals were reached and sustained over a longer period, and what lessons can be learned of relevance for funders of healthcare services.

## Methods

This is a longitudinal case study [13] of a patient choice reform in Region Stockholm.

### Study setting

Region Stockholm, previously known as Stockholm County Council, is a self-governing public authority, responsible for providing healthcare to the 2 million inhabitants of the region. A regional assembly is elected in general elections every four years. Health services are funded by the region out of regional proportionate pay-roll tax (80% of the total expenditure), complemented by patient co-payments (10%) and policy-related, direct government subsidies (10%). The regional assembly appoints a regional government, which manages the region-owned healthcare providers (hospitals and specialised care, primary care, rehabilitation, and ambulance services) (“the owner”). In addition, the assembly appoints a healthcare services committee of regional politicians and allocates the total health services budget to the committee. The committee and its administrative office form the “purchaser” of healthcare services, which contracts all public and private providers that are funded by the region.

In the early 1990’s, the region introduced a “purchaser-provider split” by establishing the organisation described above. An internal market was created. The purchaser signed cost-and-volume contracts with the public providers, with some policy-guided incentive schemes, and citizens were then given the right to choose their own primary care provider. In 1999, a conservative regional government privatised one of the region-owned acute care hospitals and signed a ten-year contract with the new owner. In the early 2000’s, it sold-off primary care centres, mainly by offering publicly employed staff to “take-over” their work-place at favourable conditions. Later, many of these provider organisations were acquired by private investor-owned corporations. Presently, 60% of the publicly funded primary care services are provided by private enterprises.

A conservative national government established in 2008 a “retail” citizen choice system for public services as an alternative to awarding “wholesale” contracts to providers by public procurement. The system is mandatory for primary care services since 2010. Region Stockholm decided to apply this patient choice system to a number of healthcare services, including specialty services, in addition to primary care. One of the first was elective hip and knee joint replacement surgery, introduced in 2009 (“OrthoChoice”).

### Data collection and analysis

The authors were commissioned by Region Stockholm in 2010 to assess the introduction of OrthoChoice. Data was collected from orthopaedic surgery providers participating in the OrthoChoice reform. Multiple data sources were used, including administrative data, interviews, and documents.

Administrative patient level data were collected, covering two years before and two years after the procedure for all, two years prior to the introduction (years 2008–2009) and two years after the introduction (2012). Additional procedure specific data and PROM (patient-reported outcome measurement) data was acquired from the Swedish Hip Replacement and Knee Arthroplasty Registries. Complication rates pre- and post-reform were standardised for comorbidities (data source: Region Stockholm Health Database) and socioeconomic characteristics (source: Statistics Sweden). Details on data acquisition, data management and statistical analyses can be found in the internal evaluation report to Region Stockholm (https://ki.se/en/lime/clinical-management see Reports). Follow-up data on annual data on patient volumes, waiting lists, and costs covering years 2013–2019 were provided by the regional purchasing agency.

Information on changes in business and clinical practices was collected by interviewing 11 clinical managers representing provider organisations in 2010. Follow-up interviews were performed in 2014 (6 clinical managers and 13 healthcare staff) and in 2017 (9 clinical managers and 6 staff). Semi-structured interview guides were used with open-ended questions. Managers and clinical staff were purposively selected based on the expectation that they would be knowledgeable about how the patient choice reform had influenced business and clinical practices. Participants were contacted by email and follow-up phone calls. Informed consent was obtained from participants. Interviews, 45 in all, were digitally recorded and transcribed verbatim.

Interviews in 2010 and 2017 were analysed by conventional content analysis [14]. Meaning units were identified, coded, and grouped into categories and themes. The 2014 interviews were originally collected for another study [15], but was subjected to a secondary analysis using a directed content analysis [14], guided by the findings from the 2010 and 2017 interviews.

Information about the patient choice scheme was for the most part obtained from the “rule book” of the Stockholm Region (https://vardgivarguiden.se/globalassets/avtal/vardavtal/vardval-stockholm/hoft%2D%2Doch-knaprotesoperationer/regelbok-hoft-knaprotes-2018.pdf.) that described the criteria regarding professional competence, physical care environment, and financial solvency that providers had to meet in order to be “authorised” and obtain permission to receive patients under the patient choice scheme.

The qualitative and quantitative information collected was condensed into a case description. Guided by the general knowledge of the patient choice reform, nationally and regionally, among the authors, and the themes identified in the interviews, the case analysis summarises key events, observed effects related to original policy goals, and formulates tentative explanations for these findings.

## Case description

Stockholm politicians were concerned with long waiting lists for elective surgery as well as problems with access to primary care. There were national standards on maximum waiting times (“care guarantees”) and financial rewards for meeting waiting time targets. A change in legislation enabled regions to adopt patient choice as a way of commissioning services, including private providers. Stockholm was an early adopter of patient choice in primary care, and subsequently expanded patient choice for selected elective procedures in specialised care. In 2009, hip and knee joint replacement surgery was included in the scheme. The objectives of this “OrthoChoice” reform were to eliminate waiting times and control costs and to create incentives for providers to increase productivity and improve quality. As one administrator at the purchaser expressed it, “*We want to buy quality, not services*”. Patients, having received a referral for surgery (usually from a primary care physician), now were free to select among authorised providers.

In order to be accredited to receive patients, a provider – public and private alike – had to seek authorisation from the purchaser. The detailed criteria which had to be met were published by the Agency in a “rule book”. These criteria included regular reporting of quality indicators, a minimum of 50 operations per year per surgeon,^1^ and strict requirements related to the operating rooms and implant selection. The new scheme was restricted to patients with few comorbidities that were associated with functional limitations (American Society of Anesthesiologists ASA Physical Status Classification System, categories 1 and 2^2^), totalling approximately 78%of all complete hip and knee replacement patients in 2009. Neither fractures nor fracture repair failures were covered by the model. ASA category 3 and 4 patients as well as reoperations were included in hospital contracts and reimbursed using DRG^3^ prices.

The new scheme consisted of two parts: Opening up market for private providers who could then compete with region-owned providers for patients, and introducing a fixed price scheme for the total patient episode, known as a “bundled payment”. All providers, public and private, were reimbursed according to the same bundled payment rate. The reimbursement formula contained three components:

1. “Package price”: The orthopaedic surgery providers received a package price of SEK 56,300 (€ 5600) for the continuum of care, including all diagnostic services, surgery with follow-up care, prosthetic costs, the necessary pre-surgical and post-surgical visits and in-hospital rehabilitation. Individual price adjustments were deemed unnecessary as patients form a homogenous group. To ensure that the initial diagnosis occurred in primary care (and that the cost of the medical work-up was born by that primary care centre), compensation for initial visits were reduced by 50% if more than 35% of referred cases were not in need of surgery.
2. “Complication warranty”: Providers were held responsible for any complications and became financially responsible for complications that occurred within two years after the arthroplasty and demanded in-hospital care or reoperations. If the patient was treated for a postoperative infection, the warranty was extended to five years, as infections increase the risk for a reduced “life-expectancy” of the prosthesis.
3. “Performance-related pay”: As part of a general policy of the region, 3.2% of reimbursement was withheld and then paid at the end of the year if providers achieved pre-defined quality targets.

When patient choice for hip and knee replacement surgery was introduced in 2009, five public and five private providers applied and were granted accreditation. The public providers were acute care multi-specialty hospitals with established orthopaedic surgery programmes. The private providers included one hospital and four free-standing centres specialised in orthopaedic surgery, established as “focused factories” for hip and knee replacements.

An initial follow-up evaluation compared data between 2008 (one year prior to the reform) and 2011–2012 (one and two years after the reform). The total number of operations, including those for ASA 1–2 patients covered by OrthoChoice, and those for ASA 3–4 patients cared for by public hospitals only and covered by another payment scheme, rose from 3721 to 4315 (+ 16%) and ASA 1–2 cases stabilised at 3000 annually. The average cost to the purchaser of a patient episode was reduced by 14%. Total costs decreased from SEK 269 m to SEK 259 m (− 3%). The proportion of patients waiting more than 90 days for their operation decreased from 33 to 13%. Providers reported that patients could choose a date of their convenience. Consequently, those with long waiting times were patients with co-morbidities or complicated conditions waiting for admission to an acute care hospital (and not covered by the choice scheme) or those who deliberately chose to wait.

Operational efficiency gains were assessed by analysing mean length of stay for the whole care episode including post-operative in-hospital rehabilitation and the average daily number of operations per operation theatre and team. The former decreased from 6.7 days to 5.8 days, a result reached after adjusting for a secular trend, gender, and type of surgery. The latter increased from 3.1 to 3.7 procedures per theatre per day.

The effect on outcomes was measured as risk of complications, adjusted for age, sex, co-morbidities, and socioeconomic characteristics of patients. The two-year complication risk decreased by 16% and the risk of reoperation by 24%, both statistically significant improvements.

No controversies concerning the complication warranty arose during the follow-up period. In all cases the medical advisor at the purchasing agency negotiated settled agreements with the providers.

Patient reported pain before and after the operation, measured using a visual analogue scale (1–100), did not change during the four years of follow-up, having an average of 62 before the operation, and 14 after the operation. The evaluation team assessed this as indicative that no “indication shift” had taken place, i.e. a tendency to operate on patients with less severe disease.

Hip and knee replacement surgery volumes remained stable after the years of close follow-up. The total numbers and the numbers of choice patient operations annually in 2013–2019 were 4442 (of which 3035 in the patient choice scheme), 4364 (2900), 4192 (2737), 4793 (3228), 5614 (3696), 5562 (3924), 5647 (4130).

The bundled payment was originally set at SEK 56,300 and was adjusted in years 2013, 2018 and 2019. The 2019 reimbursement rate was SEK 60,757.

## Analysis

The Stockholm Region introduced patient choice for providers of elective hip and knee replacement surgery to compete for patients with referrals from primary care. They accredited those providers, including private providers, that met prerequisites stated in the “Rule book”. A price was fixed for the full patient-episode (“bundled payment”), and providers were held clinically and financially accountable for complications. The stated policy goals were to improve access to care, control costs, and increase quality. Waiting times were, in practice, eliminated. Case volumes increased initially, eventually stabilising for some years and have now increased during the last years due a growing population with more elderly citizens. The initially higher volumes were achieved at a reduced total cost to the region. Complications decreased and no “indication creep” was observed. The effects in terms of increased supply of services without cost increases and quality deterioration were preserved ten years after the reform.

In the following, we explore how care providers interpreted the new scheme by analysing views of purchaser and provider representatives.

We identified six themes that capture key actor views that were consistent over the entire follow-up period.

### Theme 1: the design of OrthoChoice

The interviewees described the patient choice scheme in positive terms, explaining that its intention was aligned with their professional ambitions to reduce waiting times and suffering. However, they took issue with a number of points in the design.

The ASA classification, used to define which patients were included in the choice system, was seen as a blunt instrument which did not predict the actual costs of care. Age and possible lack of social support were factors that were not part of the classification, but which could prolong the post-operative phase.

Patients entered OrthoChoice through referrals from primary care. Providers expressed frustration over the quality of the referrals they received and that they had to rely on primary care to conduct the initial assessment as they were punished financially if a referred patient did not meet indications for surgery. Yet, if they returned poor referrals, they risked losing the patient to another provider. They were also worried primary care physicians would select a different provider for future referrals.

Providers felt that the initial process of setting the bundled payment price by the region was not transparent. The region was reluctant to initially discuss the price and later to make adjustments. Private providers had to admit patients from other regions and private patients in order to secure profitability. While accepting the complication guarantee as a basic feature of the bundled payment, some providers felt that a safeguard in the form of an additional compensation for each case would make it easier to absorb the costs of possible complications (“stop-loss”). Others suggested a special fund be set aside to pay for complications and that an upper limit on the healthcare provider’s liability for complications should be introduced.

### Theme 2: strategies developed to adjust to OrthoChoice

Five out of ten surgical units, all private, made changes in clinical processes and work organisation.. Those included process mapping and standardisation with introduction of manuals and checklists for staff. In addition, staff had to undergo an assessment of their professional qualifications in order to be “certified” to perform or assist in the operations. Study visits were made to competitors both nationally and internationally. Some private providers focused on patient segmentation, marketing, and improving patient flow also.

The public hospital providers, many with university affiliations, did not make significant changes. They continued to receive, in addition to OrthoChoice patients, patients in ASA categories 3 and 4, i.e. complicated cases and patients with co-morbidities that provided them with a different per case funding (based on DRG rates). The hospital orthopaedic surgery departments had the responsibility to care for all types of patients in their specialty, with the less time and resources to focus on one patient group only.

Providers separated acute and elective patient pathways, in some instances as a response to the OrthoChoice reform, while others had already reorganised. Some providers worked to match capacity with demand so that their booking procedures ensured patients could be managed using available resources. A number of strategies to improve OR efficiency was employed. These included scheduling a set number of operations per day with set time limits, using experienced surgeons who could operate within those time limits, and standardising clinical procedures and choice of prosthetics. Surgery was booked directly after the first visit to minimise the need for rescheduling. The “handbook” required improvement in wound management with standardised follow-up telephone calls. More effective pain management made it possible to discharge patients earlier.

Private providers, in particular, developed strategies to actively attract patients. Websites provided information on waiting times, patient satisfaction, and risks associated with operations, and potential patients received much more detailed information than required by the purchaser. Private providers also focused on promoting recognition of their brands through social media or traditional marketing. Public providers organised “open house meetings” for potential patients and patients that already had “enrolled” with the provider in order to enable clinical staff to establish personal contacts. Engaging patients in an “Arthrosis School” was also seen as an opportunity to develop a personal relationship which encouraged patients to choose that provider, made it easier to tailor the planned care process to patients’ specific needs, and empowered patients to be active participants in their own care.

To attract and retain staff was deemed crucial by all providers. Private providers introduced certification to ensure their staff had proper training for the procedures. Competition for skilled orthopaedic surgeons turned out to be more important than competition for patients. Skilled surgeons were not only instrumental to good outcomes, they also attracted patients by reputation.

### Theme 3: effects of OrthoChoice

A majority of providers were positive to the introduction of OrthoChoice and defined it as a manifestation of patient-centredness. Providers identified four areas affected by the OrthoChoice reform.

Firstly, positive effects on quality were documented in patient administrative data and quality registries.,

Secondly, waiting times were reduced and access to care improved.

Thirdly, providers were differently affected by OrthoChoice as to patient mix. The private providers, with the exception of one, worked exclusively with OrthoChoice patients. This created a shift in patient-mix where public hospitals took care of more complicated cases, which was first perceived as a positive change since acute care hospitals are better equipped for complicated cases. That perception later shifted and public providers began to feel that private providers were “gaming the system” since they could focus on ASA 1–2 patients only, whereas public hospitals had the responsibility to admit all types of orthopaedic surgery patients. In addition, public hospitals had the additional responsibility for teaching medical students and residents and were engaged in clinical research. Others saw OrthoChoice patients, particularly those from other regions, as a way to balance costs for more complicated patients and to provide case variety for staff in training.

Fourthly, staff satisfaction, as measured in surveys, had generally improved. Concerns were expressed related to the effects on medical education and residency training, as private providers have no educational obligations. With fewer of OrthoChoice patients, public providers had fewer uncomplicated cases available for residents, which was described as a potential threat to future availability of competent surgical staff.

### Theme 4: interaction

Central to OrthoChoice was the requirement that patients should be referred by a primary care physician. Over time, the primary care centres and physicians were regularly courted by providers through the distribution of information material and meetings. One private provider involved referring primary care physicians in a shared process to ensure the safe discharge of patients, a practice that was not taken up by other providers. Providers acknowledged that there was a need for collaboration rather than competition, and a support forum was established for all organisations involved in OrthoChoice.

### Theme 5: economics of OrthoChoice

Providers unanimously stated that the fixed reimbursement for OrthoChoice patients was too low, and subsequent price adjustments were insufficient. Provider representatives claimed considerably higher unit costs, although no provider could provide detailed information on how those estimates had been calculated. Private providers improved productivity by increasing the number of operations per day. Public providers compared the reimbursement to the higher DRG rate they had previously received and reported higher total costs due to a change in patient-mix as an effect of OrthoChoice.

### Theme 6: compliance to the “rule book”

All providers reiterated that they paid close attention to the regulations set by the purchaser and included in the “Rule Book”. They meticulously followed the patient protocol, referral procedure, initial visits, indications for operations and surgical procedures, infection control, post-operative rehabilitation, and follow-up. Quality indicators and complications were reported to the quality registries as required.

## Discussion and conclusions

We have described a case of an NPM inspired approach by a self-governing public regional health authority (“the region”) with the legal obligation to provide its inhabitants with need-based equitable health care services and funding those services almost entirely with regional tax income. The scheme was twofold: 1. Introduce the right for patients in need of a knee or hip replacement to choose their service provider among public or private providers, authorised by the region; and 2. Paying providers a fixed price covering the whole patient care episode (bundled payment). We have reported that the stated policy goals of improving access to care and improving care quality, measured as declining complication rates, while containing total costs for the region were reached and sustained in the long-term.

Based on repeated interviews with purchasers and providers over a seven-year period, we found that providers, both public and private, accepted the new arrangement, and adjusted their activities to match the requirements. This was the case regardless of dissatisfaction with some of the rules introduced, e.g. patient selection criteria, the role of primary care, perception of a too low compensation, and the almost unrestricted responsibility of providers for possible complications. There were differences in strategic responses among public and private providers – the latter focusing on streamlining clinical processes and active marketing whereas the former noted a shift in patient mix towards more complicated cases (not included in the scheme) and worried about the economic ramifications.

Despite these differences in strategies, conclusions regarding differences as to financial performance between private and public providers could not be drawn, as providers were not requested to report actual cost data to the purchasers, and annual financial statements did not include information of the profitability of service lines. But figures on operational efficiency (OR capacity use) did show that private focused factories were at an advantage, in line with the observations of Kruse et al. [17] on cataract surgery, but not generalisable across procedure categories [18]. The results also showed greater responsiveness to the incentive structures of the model among private providers, a finding also reported by others [19].

The creation of provider competition was one of the stated policy goals. But rather than a focus on competition for patients, the main competition turned out to be competition for orthopaedic surgeons. This led to more experienced surgeons focusing on a few procedures, possibly one explanation of the quality gains. - On the whole, collaboration was valued higher than competition by the involved organisations. By having strict criteria for market entrance (the accreditation process), issuing detailed rules for organising care, and introducing a fixed non-negotiable price, the region was both a market-maker and market-manager. Providers adapted to this strict regulation and became highly compliant to the rules.

The strict cost control achieved in the OrthoChoice scheme was to be expected. Fixed payments for care episodes were introduced by the US Medicare system in the 1980’s with similar effects. In 2013, Medicare introduced a voluntary Bundled Payment for Care Improvement (BPCI) demonstration project with four different bundled payment models. A study focusing on lower extremity joint replacement comparing over 60,000 procedures performed between 2011 and 2015 showed that participants in the initiative had significantly lower Medicare per-episode reimbursements than non-participants, with no differences in quality of care [20]. Medicare introduced another scheme under the BPCI initiative in 2016: the Comprehensive Joint Replacement (CJR) programme that keeps providers financially accountable for both quality and costs covering a 90-day post-operative care episode. Several reports have shown effects of the CJR in terms of cost reductions and higher quality of care, as cited by Sullivan et al. [21]. Those observations are similar to previous experience of bundled payments, and to extended interventions of bilateral total joint arthroplasty [22].

Interest in bundled payments rose with the introduction of “Value-Based Health Care” (VBHC) by Porter and Teisberg in 2006 [23]. The authors suggested six strategies in their “value agenda”, and the OrthoChoice example illustrates the measurement of the outcomes and costs of the “value equation” and the strategies of organising care as integrated practice units based on medical conditions over “the full cycle of care”, and reimbursing providers with bundled payments. Although the interest in VBHC was widespread, it was also fleeting and the patient choice model was, in practice, more rooted in politicians and providers understanding of New Public Management.

Despite the considerable literature on bundled payments, as referred to above, less is known about how well providers adhere to strict regulations when competition is introduced into publicly funded healthcare markets. Above all, studies on the long-term effects have been missing. In the case that we are reporting, the public regional health authority applied a strict regulation of the market, limited to a few elective surgical procedures (hip and knee arthroplasties included), and introduced a reimbursement scheme that matched the criteria later proposed by the EU Commission appointed Expert panel on effective ways of investing in health. That panel concluded: “Where there is adequate information about quality of care and dominant positions are absent, economic theory suggests competition will force organisations to be more efficient and innovative and may therefore reduce prices. If prices are regulated and quality is observable as well as used to guide demand, economic theory predicts competition to improve health service quality” [24].

To summarise, the parallel introduction of patient choice leading to provider competition and bundled payment for a specialty service took place within a public health authority solely funding (with the exemption of minor patient charges) all healthcare services to its population (context). Using the power of the purse the health authority controlled the entrance to a strictly regulated market in an authorisation process defining requirements on providers as to professional standards, quality and financial viability. It unilaterally set a fixed price for the patient episode, equal to all providers, thus setting the scene for competition on quality. It kept providers responsible for avoidable complications (circumstances).

This governance approach might be worth emulating not only in tax-funded healthcare systems where national and regional governments hold the purse, but also increasingly in social insurance-based systems like those in continental Europe. An insurer that moves from reimbursing all providers working among the insured population to selective contracting, could well use an authorisation system that grants market entrance only to providers that fulfil strict requirements on financial viability, adequate staffing, professional and quality standards and compliance with clinical care guidelines. By setting a fixed price for a clinical service and promoting competition by allowing patients to choose providers, total cost savings driven by competition can be either kept by the insurer or shared with providers as incentives for continuous service and quality improvement.

Our conclusion is that creating competition by introducing patient choice, accepting both public and private providers, and setting the scene for competition on quality, not cost through a fixed price funding formula, in a publicly funded system, succeeds in meeting the original policy goals (improved access, quality and reduced cost). This quasi-market allows providers to make strategic choices in terms of patient segmentation and increases all providers’ focus on quality (as expected). Negative consequences might be an increased burden on public providers with the responsibility to care for complicated cases, and challenges for medical education and residency training. For once, theory seems to fit well with reality.

## Data Availability

The datasets used and analysed during the current study are available from the corresponding author on reasonable request.

## Abbreviations

NPM: New Public Management
ASA: American Society of Anesthesiologists (Physical Status Classification System)
DRG: Diagnosis Related Groups

## Footnotes

1 Most studies on the relationship between surgeon volumes and clinical quality cite 50 as a sufficient threshold of ensuring quality in total hip and knee replacement [16].

2 https://www.asahq.org/standards-and-guidelines/asa-physical-status-classification-system

3 DRG: Diagnosis-Related Groups
